# Electrically Programmable Terahertz Diatomic Metamolecules for Chiral Optical Control

**DOI:** 10.34133/2019/7084251

**Published:** 2019-02-27

**Authors:** Longqing Cong, Prakash Pitchappa, Nan Wang, Ranjan Singh

**Affiliations:** ^1^Division of Physics and Applied Physics, School of Physical and Mathematical Sciences, Nanyang Technological University, Singapore 637371, Singapore; ^2^Centre for Disruptive Photonic Technologies, The Photonics Institute, Nanyang Technological University, 50 Nanyang Avenue, Singapore 639798, Singapore; ^3^Institute of Microelectronics, 11 Science Park Road, 117685, Singapore

## Abstract

Optical chirality is central to many industrial photonic technologies including enantiomer identification, ellipsometry-based tomography, and spin multiplexing in optical communications. However, a substantial chiral response requires a three-dimensional constituent, thereby making the morphology highly complex to realize structural reconfiguration. Moreover, an active reconfiguration demands intense dosage of external stimuli that pose a major limitation for on-chip integration. Here, we report a low bias, electrically programmable synthetic chiral paradigm with a remarkable reconfiguration among levorotatory, dextrorotatory, achiral, and racemic conformations. The switchable optical activity induced by the chiral conformations enables a transmission-type duplex spatial light modulator for terahertz single pixel imaging. The prototype delivers a new strategy towards reconfigurable stereoselective photonic applications and opens up avenues for on-chip programmable chiral devices with tremendous applications in biology, medicine, chemistry, and photonics.

## 1. Introduction

Chirality is ubiquitous in nature from human hands to chemical and biological macromolecules and climbing plants. Intense research efforts have been devoted to the study of chirality to explore pharmacological effects and even unravel the origin of life [1]. In pharmaceutical industry, it is essential to differentiate the handedness of enantiomers, as it is strongly associated with their potency and toxicity [2]. Currently, circular dichroism (CD) is a routine optical approach to measure chirality [3]. However, the CD response of natural enantiomers is extremely weak, typically in the range of millidegrees and hence large volume (submillilitre) of analyte is required with a long integration time (~30 min) to precisely resolve the chirality [4]. Several solutions have been reported to overcome the detection limit of naturally weak CD response by integrating with chiral metamaterials [5–10]. An ingenious sensing scheme by virtue of synthetic chiral metamaterials could lead to a better detection accuracy of enantiomer handedness. Synthetic chirality is significant not only for the determination of enantioselective activity in biochemical reactions, but also for exceptional light-manipulation capabilities, including polarization control and detection [11–13], negative refractive index [14], perfect lens [15], and color-tunable polarizer [5]. Metamaterials, with artificially constructed building blocks, enable extraordinary chiral optical responses and, moreover, provide the freedom to actively reverse the polarity of chirality.

Since chirality is inherently a three-dimensional (3D) phenomenon that requires coupling between the local electric and the magnetic dipoles [16, 17], artificial constituents are usually designed with complex conformations [18], such as spiral with multiple helices [11, 16, 19], or near-field coupled multilayer metamaterials [20, 21]. Polarity reversal of intrinsic chirality is usually accompanied by 3D structural reconfiguration of metamaterial constituents via various approaches [22–24], such as by integrating semiconductors (silicon) [18], phase change materials (Ge_2_Sb_2_Te_5_) [25], graphene [26], magnesium [24], DNA scaffold [27], and MEMS actuators [11, 19]. However, a structural reconfiguration usually demands intense stimuli, like large dosage of optical pump fluence (~1 mJ/cm^2^) [18], high voltage (~350 V) [19], high temperature (180°C) [25], chemical reaction (hydrogenation) [24], or pneumatic control (10 Pa) [11] that limits the applications of these fascinating devices. Although electrostatically actuated bimorph cantilevers have exhibited excellent performance in a plethora of interesting applications in reconfigurable photonic devices [28–36], it is still challenging to achieve structural handedness reversal of chiral constituents with* weak* electrical stimulus. So far, small dosage of external stimulus has failed to realize an effective chiral polarity reversal, let alone building more sophisticated chiral conformations.

In this article, we report an electrically programmable chiral paradigm actuated by 10 V electrical bias that is reconfigurable among full chiral conformations including* dextrorotatory* (*D*),* levorotatory* (*L*), and* racemic* (*r*) and* achiral* (*a*) states. The unique diatomic metamolecules enable both intrinsic and extrinsic chiral optical responses that are manipulated via dual-band resonance excitation. The switchable optical activity induced by the chiral reconfiguration reveals good capacity of high-speed and high-quality polarization rotation. We demonstrate a duplex spatial light modulator utilizing the polarization-dependent intensity modulation for terahertz single pixel imaging with a faster frame rate by virtue of the dual-band chirality.

## 2. Results

### 2.1. Design of the Reconfigurable Chiral Scheme

**Figure 1:**
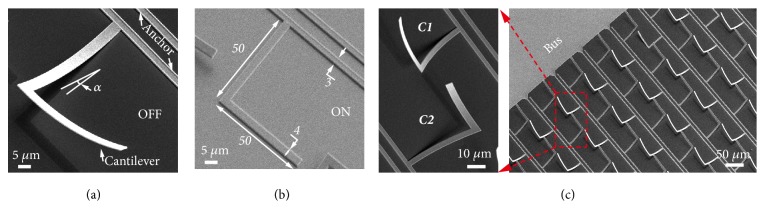
*MEMS chiral device configuration.* (a, b) SEM images of one bimorph microhelix in suspended state (defined as OFF, 3D configuration) and actuated states (ON, 2D planar configuration), respectively. Geometrical parameters of anchor and cantilever are indicated, and thickness of metal (aluminum, *t*_Al_) in the bimorph cantilever is 400 nm. (c) Scheme of the metamaterial array comprising of diatomic metamolecules with microhelices of opposite handedness. A zoomed-in image of a metamolecule is highlighted where two microhelices are electrically isolated and separately connected to two actuation channels (C1 and C2).

**Figure 2:**
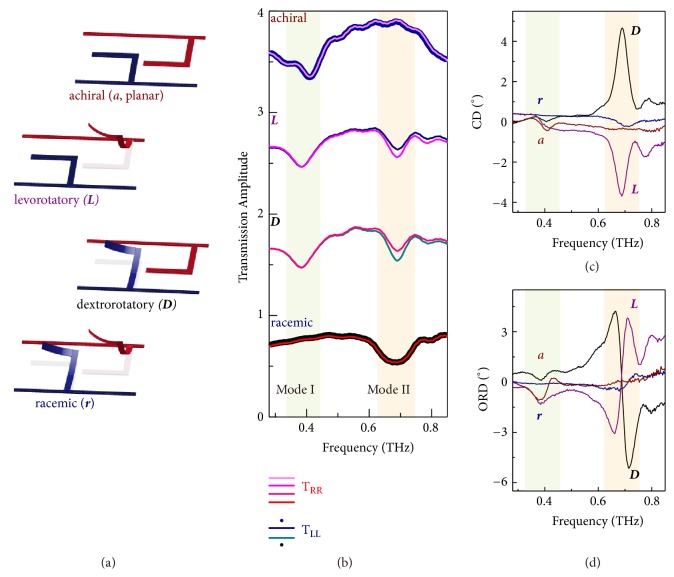
*Chiral conformations and experimental optical responses.* (a) Schematic diagram of four chiral geometries in one metamolecule with achiral (*a*, planar conformation), levorotatory (*L*), dextrorotatory (*D*), and racemic (*r*) conformations. (b) Experimental copolarized transmission amplitude of the metamaterial at the four conformations with RCP and LCP light at normal incidence. The RCP and LCP spectra reveal identical responses for* a*- and* r*-conformations, and spectral difference emerges at mode II at* L*- and* D*-conformations. (c,d) Circular dichroism and optical rotatory dispersion spectra at the four different chiral conformations.

Here, we utilize an L-shape bimorph cantilever that self-assembles as a 3D microhelix together with a fixed anchor after the release process (see Figure 1(a), Methods and Supplementary [Supplementary-material supplementary-material-1])[29]. The bimorph cantilever comprises metal (aluminum, Al) and dielectric (alumina, Al_2_O_3_) layers, and residual stress between the two materials gives rise to the bending force that is utilized for assembly of a microhelix. Such a microhelix possesses intrinsic chirality (Supplementary [Supplementary-material supplementary-material-1]) determined by its handedness, which vanishes when it is pulled down onto the substrate forming a planar resonator. A straightforward way to reconfigure the microhelix is by applying a pull-in voltage across the metal (Al) and silicon substrate (10 V, [Supplementary-material supplementary-material-1]). The electrostatic force neutralizes the residual stress and pulls the cantilever back onto the substrate surface as shown in Figure 1(b). In order to realize a structural handedness reversal, a metamolecule comprising two microhelices with opposite handedness was conceived as shown in Figure 1(c), which is a basic building block of the metamaterial array. The two neighboring microhelices are electrically isolated from each other, which allows for independent control through two separate actuation channels (C1 and C2). For binary electrical encoding, state of microhelix without electrical input is defined as OFF (0), and 2D planar state is defined as ON (1). The independent control of dual-channel electrical inputs enables four reconfigurable states of the diatomic metamolecule with unique chiral response at each of those states. As shown in Figure 2(a), when all cantilevers are in-plane forming a 2D metasurface, the conformation exhibits an* intrinsic achiral* response [37]. When only the left-handed (right-handed) microhelix is out-of-plane in each metamolecule, it forms a* levorotatory* (*dextrorotatory*) chiral conformation with intrinsic chirality. On the other hand, when both of the microhelices are out-of-plane in each metamolecule, mixture of equal-quantity of microhelices with opposite handedness enables* racemic* conformation. The* dextrorotary *(*D*) conformation couples strongly with right-handed circularly polarized (RCP) resulting in a relatively weak transmission intensity. On the contrary, the left-handed circularly polarized light (LCP) couples weakly to* D*-conformation due to the absence of* levorotatory* (*L*) microhelix, thereby giving rise to a different transmission intensity for RCP and LCP light. This difference generates circular dichroism, which is direct quantitative evidence of intrinsic chirality.

The above interpretation is experimentally demonstrated by testing the metamaterial prototype reconfigured at the four different chiral conformations using a terahertz time-domain spectroscopy system (Supplementary [Supplementary-material supplementary-material-1] and [Supplementary-material supplementary-material-1]). In terms of* circular polarization*, the copolarized transmission spectra of RCP and LCP were measured (see Methods) as shown in Figure 2(b). At* achiral* (*a*) and* racemic* (*r*) conformations, exactly identical transmission spectra (T_*RR*_ and* T*_*LL*_) emerge indicating no chiral optical response in the far field. Although no far field chiral features can be measured at these two conformations, they possess starkly different chiral states: (1) different spatial conformations and (2) different resonance mode frequencies at 0.4 THz (*achiral*, mode I) and 0.7 THz (*racemic*, mode II). The* a*-conformation inherently does not possess any chirality while the* r*-conformation lacks chirality due to the cancellation of near-field chiral responses in the far field. The resonance frequency shift is caused by the structural deformation between 3D and planar resonators in the metamaterial that introduces change of effective capacitance [29]. At* L*- and* D*-conformations, mode I and mode II are simultaneously excited with coexistence of 2D and 3D resonators in a metamolecule. However, spectral difference only emerges in the vicinity of mode II originating from the 3D microhelix that possesses intrinsic chirality. The transmission difference indicates contrasting complex refractive indices of the chiral metamaterial for RCP and LCP incident terahertz waves [14]. The difference in the imaginary part of refractive indices generates circular dichroism (CD), while the difference in the real part of refractive indices manifests as optical rotatory dispersion (ORD) [22]. The CD and ORD are numerically defined through transmission spectra as *CD* = tan^−1^[(|*T*_*RR*_| − |*T*_*LL*_|)/(|*T*_*RR*_| + |*T*_*LL*_|)] and *ORD* = arg⁡(*T*_*RR*_) − arg⁡(*T*_*LL*_), respectively. In the coherent terahertz spectroscopic system, we measured both amplitude and phase information of the chiral metamaterial and thus probe the CD and ORD properties simultaneously as shown in Figures 2(c) and 2(d), respectively. The maximal CD value was measured to be 4.6° with* D*-conformation at mode II, which is order of magnitude higher than CD of natural* D*-enantiomers. The faint chiral responses at mode I at the four conformations mainly originate from two factors: nonradiative dissipation in the metal [38] and imperfect normal incidence in measurements [37]. The CD polarity is reversed at* L*-conformation, and no obvious CD response is captured at* a*- as well as* r*-conformations. Since CD and ORD spectra are linked by Kramers-Kronig's relation, ORD spectra manifest correlated responses as shown in Figure 2(d), where large optical activity appears at* D*- and* L*-conformations with a significant enhancement in the vicinity of mode II.

### 2.2. Modulation of Intrinsic Chirality

**Figure 3:**
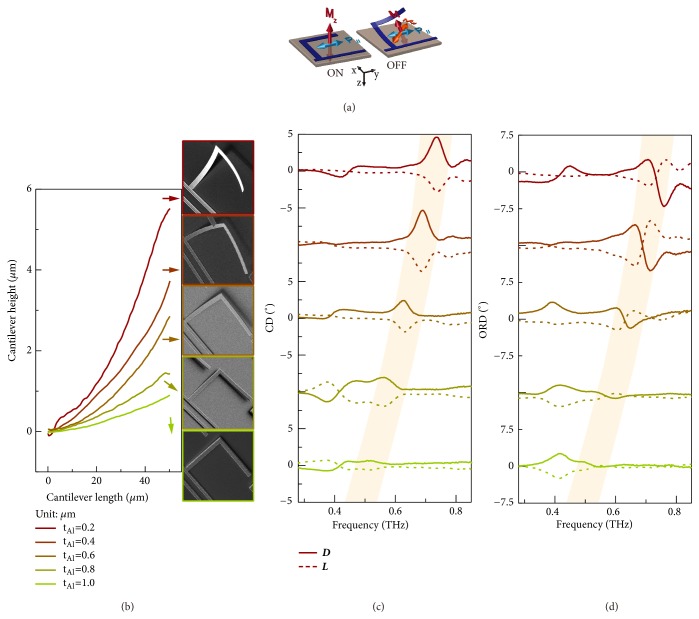
*Experimental modulation of intrinsic chirality.* (a) Schematic diagram showing the electromagnetic origin of intrinsic chirality. (b) Reflection digital holographic microscopic profiles of suspended cantilever arm that is attached to anchor with various metal thicknesses (*t*_Al_) and fixed dielectric layer thickness (*t*_d_). SEM images of microhelices with different cantilever profiles are also shown. (c, d) Experimental CD and ORD spectral responses for different samples at* D*- and* L*-conformations. A continuous modulation of intrinsic chirality strength and resonance frequency is observed.

In terms of electromagnetic interactions, a generalized condition of $\vec{p}\cdot \vec{m}\neq 0$ is required for an intrinsic chirality, where $\vec{p}$ and $\vec{m}$ are the moments of net electric and magnetic dipoles, respectively. In a 2D resonator, however, electromagnetically induced magnetic dipole is invariably vertical to the substrate surface (*M*_⊥_) with electric dipole in-plane (*P*_//_) at normal incidence (Figure 3(a)), i.e., $\vec{p}\cdot \vec{m}\equiv 0$ without intrinsic chirality neglecting nonradiative dissipation and substrate effect [37, 38]. To break the electromagnetic multipole symmetry, on the other hand, a 3D constituent has to be introduced that extends the electromagnetic interaction spatially in the third dimension and thus enables a nontrivial electric and magnetic dipole interaction as schematically shown in Figure 3(a). In this context, intensity of chiral optical response would strongly depend on the geometrical configuration of the constituent which determines the electromagnetic multipole interactions. As a typical example, the microhelix supports a current loop at mode II that is guided to flow on the 3D conformation together with the fixed anchor (see Methods and Supplementary [Supplementary-material supplementary-material-1]). This 3D distribution of oscillating current enables nontrivial electric and magnetic interactions ($\vec{p}\cdot \vec{m}\neq 0$) in the localized field and manifests as an intrinsic chirality in the far field at* D*- and* L*-conformations.

The interpretation is verified in the microhelix design which offers the flexibility to tailor the geometric configuration in the spatial* z*-direction. According to the schematic diagram in Figure 3(a), in-plane projection of magnetic dipole (*M*_//_) depends on the inherent suspension angle of cantilevers (*α, *Figure 1(a)), i.e., pitch of the microhelix. A larger pitch of microhelix would enable a stronger interaction of electromagnetic components within the scope of current design. For the bimorph cantilever, the inherent suspension angle depends on the residual stress between Al and Al_2_O_3_ layers that is numerically estimated in terms of the radius of curvature (*r*) as follows:

(1) $$\begin{array}{c} \frac{1}{r}=\frac{6n\left(1+n\right)\left(m\sigma_{Al}-\sigma_{d}\right)}{t_{Al}E_{Al}\left[K+3mn\left(1+n^{2}\right)\right]} \end{array}$$

where *K* = 1 + 4*mn* + 6*mn*^2^ + 4*mn*^3^ + *m*^2^*n*^4^, *m* = *E*_*Al*_/*E*_*d*_, and *n* = *t*_*Al*_/*t*_*d*_. Here* t*_*Al*_ and* t*_*d*_ are the thickness of metal (Al) and dielectric layer (Al_2_O_3_) of the bimorph cantilever,* E*_*Al*_ and* E*_*d*_ are the Young's modulus, and* σ*_*Al*_ and* σ*_*d*_ are the residual stress of the Al and Al_2_O_3_, respectively. Young's modulus and residual stress of materials are constant at room temperature determined by material properties and fabrication process, and* r* can be engineered by tailoring* t*_*Al*_ and* t*_*d*_. According to (1), a smaller *t*_Al_ would result in a smaller* r* at a constant* t*_*d*_ and, thus, a larger pitch of microhelix.

As presented in Figure 3(b), metamaterial devices with various metal thicknesses of cantilevers (0.2, 0.4, 0.6, 0.8, and 1.0 *μ*m) were fabricated. The deformation profiles were tested using reflection digital holographic microscope (R-DHM, Supplementary [Supplementary-material supplementary-material-1]). A larger cantilever deformation in the* z*-direction is realized with a thinner metal thickness, which is also visualized by the respective scanning electron microscopic (SEM) images of microhelices at OFF state (Figure 3(b)). We have tested the chiral optical responses of these samples at* D*- and* L*-conformations with different microhelix pitches. As revealed in Figures 3(c) and 3(d), intrinsic chirality in terms of CD and ORD spectra is modulated with a clear trend in the chiral strength as well as resonance frequency at mode II. The resonance frequency is also altered due to the modulation of effective capacitance in the microhelices at different deformation profiles.

### 2.3. Extrinsic Chirality

**Figure 4:**
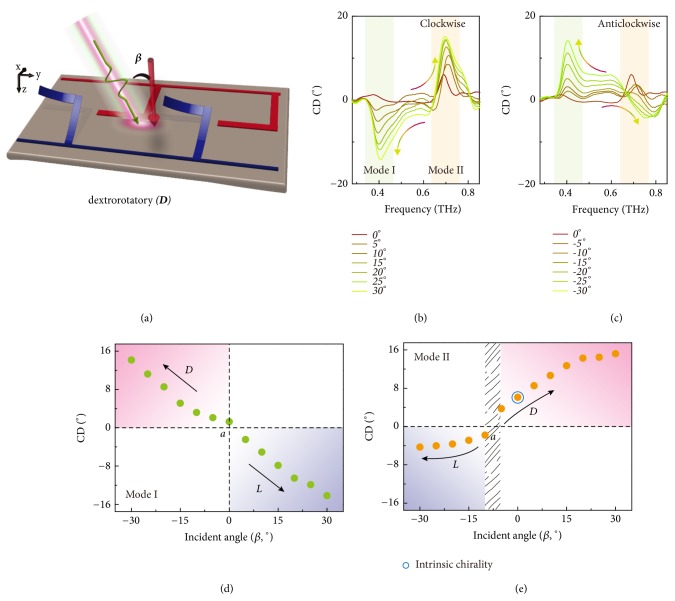
*Experimental modulation of extrinsic chirality.* (a) Schematic illustration of extrinsic chirality by tilting the incident wave vector relative to the normal of the metamaterial surface. (b, c) Experimental incident angle dependent CD responses with clockwise and anticlockwise tilt by measuring the* D*-conformation. (d, e) Modulation of extrinsic chirality at mode I and mode II. Intrinsic and extrinsic chirality reveals the same geometrical origin, and intrinsic chirality can be compensated by the extrinsic one.

Apart from the intrinsic chirality enabled by 3D conformation of microhelix, we have also probed the extrinsic chirality benefiting from the unique chiral metamolecule at* D*- or* L*-conformation with both 2D and 3D resonators. As schematically shown in Figure 4(a), extrinsic chirality is induced in a 2D resonator that lacks symmetry by tilting the incident wave vector relative to the normal of the resonator. Although extrinsic chirality has attracted a plethora of research interest that gets rid of complex 3D constituents [37], there is no clear interpretation and experimental demonstration of inherent relationship between intrinsic and extrinsic chirality. Using the* D*-conformation as a testbed, we have performed incident angle-resolved measurements and excavated the inherent relation by synchronously modulating intrinsic and extrinsic chirality. The incident angle is defined as “*β*” relative to the normal of the sample surface as illustrated in Figure 4(a), and chirality in terms of CD is measured. The CD spectra are summarized in Figures 4(b) and 4(c) by sweeping *β* from -30° to +30° with a step size of 5°, where the sign “-” indicates an anticlockwise tilt and vice versa. As expected, extrinsic chirality appears at mode I induced by the 2D resonators whose intensity is strongly dependent on *β* and chiral conformation is determined by the sign of incident angle. Interestingly, the intrinsic chirality at mode II is also strongly modulated depending on the incident angle. Moreover, the intrinsic chiral conformation becomes reversible just by tilting the incident angle. The peak value of CD is plotted in Figures 4(d) and 4(e) at mode I and mode II, respectively. An approximately linear relation between CD and *β* is revealed at mode I for purely extrinsic chirality, where the achiral point lies at the normal incidence. A clear angular dependence is also observed at mode II for mixed chirality, and the intrinsic chirality is compensated by extrinsic chirality with an achiral point lying at a* nonzero *incident angle. Due to the opposite handedness of the two resonators in a metamolecule, the extrinsic chirality reveals opposite CD parities at the same incident angle. The geometrical correlation between intrinsic and extrinsic chirality is unraveled through this unique chiral platform. Intrinsic chirality can thus be further enhanced in a uniformly periodic system by introducing extrinsic chirality, which would largely reduce the fabrication complexity, especially at nanoscale dimensions.

### 2.4. Programmable Chiral Photonic Device

**Figure 5:**
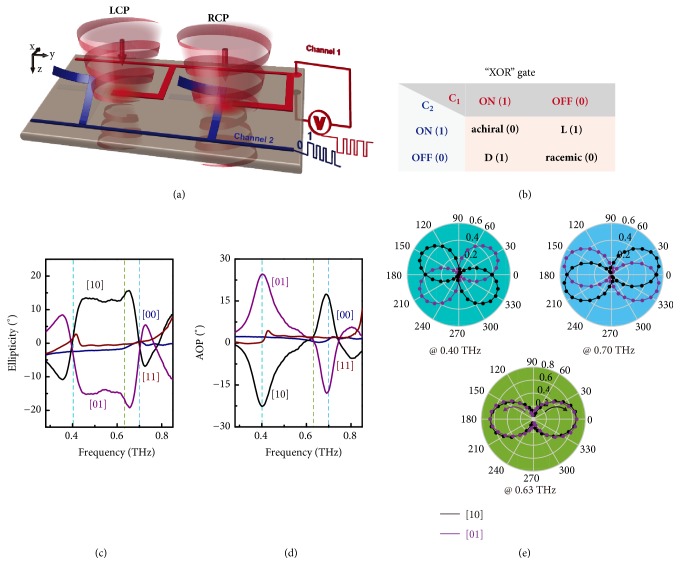
*Electrically programmable chiral platform.* (a) Schematic diagram of the electrically programmable chiral platform. The neighboring two microhelices in each metamolecule are electrically isolated and separately connected to input channel 1 (C1) and channel 2 (C2). Encoding sequences [11], [01], [10], and [00] enable the four chiral conformations:* a*,* L*,* D*, and* r*, respectively. The diagram schematically shows* D*- conformation with [10] input that gives rise to intrinsic chirality with a nontrivial CD response. (b) Summary of the logical operation for the programmable chirality platform in terms of intrinsic optical chirality output following an “exclusive OR (XOR)” gate. A nontrivial CD output is defined as “1” and a trivial CD output is defined as “0”. (c, d) Measured output polarization states described by ellipticity and rotation angle of polarization (AOP) with* y*-polarized incidence at four chiral conformations enabled by the respective encoding sequences. (e) Visualized output polarization states in polar coordinates that are measured at three specific frequencies with [01] and [10] inputs. The output polarization plane is rotated by large angles at 0.4 and 0.7 THz, and polarization state is modulated to elliptical states with opposite handedness at 0.63 THz. Dots are measured angle-resolved transmission intensity by rotating an analyzer.

The proposed simple chiral scheme has been demonstrated with abundant chiral conformations and controllable intrinsic as well as extrinsic chirality, which could thus be applied as a versatile chiral platform. As we have mentioned, residual stress in the bimorph cantilever actuates the 3D configuration of microhelix, which can be neutralized by applying a 10 V pull-in voltage so that the microhelix is reconfigured to the 2D resonator structure. Since the two microhelices in one metamolecule are electrically isolated and connected to different external sources (C1 and C2), they are actuated independently, which enables a dibit encoding capacity (Figures 1(c) and 5(a)). According to whether actuation voltage is applied, the input is defined as “1” or “0”, and thus* a*-,* L*-,* D*-, and* r*-conformations correspond to [11], [01], [10], and [00] encoding sequences, respectively. The far field chiral optical response is considered as the binary output where “0” indicates a trivial CD value and “1” indicates a nontrivial CD value. Based on the measured intrinsic chiral responses at the four conformations, the logical operation of the system is summarized in Figure 5(b), and it shows a programmable property following an exclusive OR (XOR) gate operation in terms of a dual-channel electrical input and binary chiral optical output.

One important application of optical chirality is the polarization modulation of light that is a key technology for optical communication, imaging, and polarimetric spectroscopy. Here, we also explore the programmable polarization modulation property of the chiral device. To probe the polarization conversion performance, four elements of Jones matrix (see Methods) are measured with* linearly* polarized incidence at four reconfigurable conformations with *t*_Al_ = 0.4 *μ*m (Supplementary [Supplementary-material supplementary-material-1]). The polarization spectra are numerically expressed in terms of ellipticity and angle of polarization (AOP) as shown in Figures 5(c) and 5(d). In experiments, the polarization of incident light was aligned parallel to the axis of anchor (defined as* y*-polarized incidence) in order to exclude the anisotropy induced by anchors. Output polarization state is preserved (“0” output without polarization modulation) for [11] and [00] inputs. For [10] and [01] inputs, strong polarization modulation (“1” output) occurs in a broadband frequency regime extending from 0.4 to 0.7 THz (mode I and mode II). The output polarization state is modulated from linear to elliptical states with opposite handedness, and the ellipticity attains a value of ~±15° for [10] and [01] inputs, respectively. In addition, strong optical activity is observed at 0.4 and 0.7 THz, where linear polarization plane is rotated by up to ±25° with linear polarization state preserved at 0.4 THz for [01] and [10] inputs, respectively. The opposite handedness of microhelices in a diatomic metamolecule enables reversed rotation angles at 0.4 and 0.7 THz at a specific conformation [39].

In order to visualize the output polarization contrast between [10] and [01] inputs, polarization states in polar coordinate at three specific frequencies were measured in terms of transmission intensity as plotted in Figure 5(e) (Supplementary [Supplementary-material supplementary-material-1]). At 0.4 and 0.7 THz, linear polarization states are preserved, but polarization planes are rotated by ∓25° and ±17° for [10] and [01] inputs, respectively. At 0.63 THz, linear polarization state is transformed to elliptical states with opposite handedness without polarization plane rotation for [10] and [01] inputs (Supplementary [Supplementary-material supplementary-material-1]). All three polarization conversion patterns enable large output polarization contrast between* D*- and* L*-conformations, which could provide solution to rapid polarization-division multiplexing in high-speed optical communication and real-time polarization-dependent imaging. In terms of optical output on polarization modulation, the device also follows an XOR gate logical operation. The switching time of the bimorph cantilevers is estimated to be ~2 microseconds as determined by the mechanical resonance frequency (Supplementary [Supplementary-material supplementary-material-1] and [Supplementary-material supplementary-material-1]). Similar to the chiral response, the polarization modulation performance can be tailored by varying the metal thickness of the cantilevers (Supplementary [Supplementary-material supplementary-material-1]).

### 2.5. Spatial Light Modulator and Single Pixel Imaging

**Figure 6:**
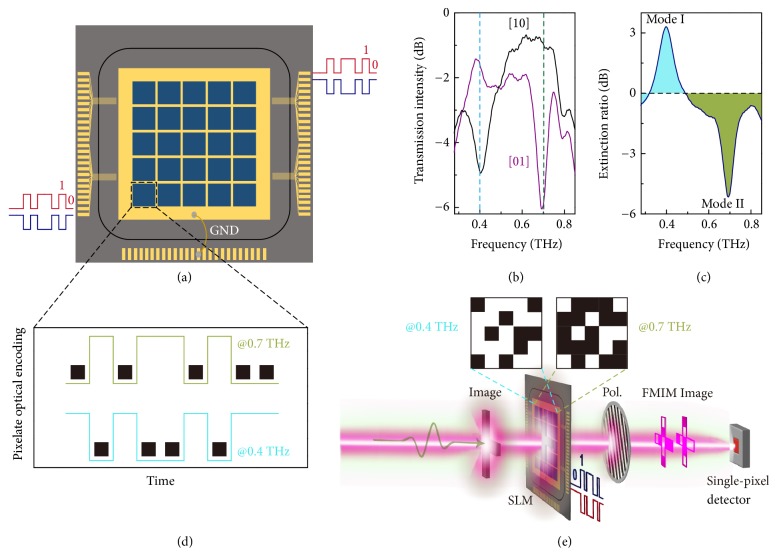
*Application as a FMIM SLM and single pixel imaging.* (a) Schematic diagram of the 5×5 pixelate terahertz spatial light modulator operating in the transmission mode. Each pixel is identical and electrically isolated to be encoded separately. (b) Experimental transmission intensity contrast of light. Large intensity contrast occurs at mode I and mode II through the pixel with [10] (*D*-configuration) and [01] (*L*-configuration) encoding sequences with a wire-grid polarizer oriented at 20° relative to* y*-axis. (c) Optical intensity extinction ratio spectrum between the two operation states of each pixel. Significant extinction ratios (>3dB and > -5dB) enable excellent SLM performance, and opposite polarities at different frequencies provide a frequency-division multiplexing channel which multiplies the data capacity. (d) Digitalized optical output according to the output light intensity. ■ indicates a relative low intensity output from a single pixel. The two frequency-divided output channels are correlated with opposite output signals. (e) Schematic diagram of optical setup for terahertz single pixel imaging using the frequency-division multiplexed intensity modulated (FMIM) SLM as a core coding component.

The significant output polarization contrast between* D*- and* L*-conformations leads to a giant optical intensity modulation by introducing a wire-grid polarizer (WGP), which allows loss-free transmission of light with one polarization state but attenuates the light intensity of orthogonal polarization state. This electrically encoded chiral platform can thus function as an excellent intensity modulation module for an all-solid terahertz spatial light modulator (SLM), as schematically shown in Figure 6(a). As a proof of concept, we propose a SLM of 5×5 pixels, where each pixel is electrically isolated and encoded separately to provide a real-time spatial intensity modulation of light. All pixels are identical and composed of arrays of the diatomic chiral metamolecules. The polarization-dependent transmission intensity spectra of the module are measured after a WGP oriented along 20° relative to* y*-axis (that is, linearly polarized light of 20° relative to* y*-axis transmits without loss) as shown in Figure 6(b). In this experiment, we utilized the metamolecule with configuration of metal thickness *t*_Al_ = 0.4 *μ*m as the SLM module, which enables ~±20° polarization rotation at mode I and mode II (Figure 5(d)). With the WGP angle fixed, the output light intensity is strongly modulated between [10] (*D*-conformation) and [01] (*L*-conformation) states at 0.4 THz as well as 0.7 THz, respectively. The intensity extinction ratio reaches more than 3 dB and -5 dB at these two modes (Figure 6(c)). More interestingly, the extinction ratio reveals opposite polarities at mode I and mode II due to the opposite polarization states at 0.4 and 0.7 THz, which enables the simultaneous light modulation at two frequencies with opposite intensity outputs. This frequency-division multiplexed intensity modulation (FMIM) technique is commonly applied in optical communication system. In the SLM scheme, the digitalized optical output intensity of each single pixel is encoded by input electrical signals, where relatively larger intensity of light is regarded as “1” and the smaller intensity of light is regarded as “0” (also visualized as a dark pixel ■), as indicated in Figure 6(d). With the real-time spatial encoding of transmitted light by each localized pixel, large amount of information could be loaded for communication and imaging. The opposite polarities at two frequencies lead to different FMIM spatial light patterns and thus multiply the information loading capacity.

This SLM is a core component to develop imaging modality at long-wavelength regime, especially at the terahertz frequencies. Although isomorphic mapping of objects to the focal plane arrays is conventional in visible, large density detector array is usually unfeasible at longer wavelength. An alternative is the single pixel imaging with detector of only one pixel, and the image is spatially encoded by various SLM masks into a matrix (*M*×*N*, where* M* is sequential measurements and* N* is elements of image), which is then inversed to reconstruct the original image [40], as schematically illustrated in Figure 6(e). Several spatial multiplexing approaches have been developed for the measurement matrix, like raster-scan (commonly adopted in television display), random, and Hadamard matrices [41]. The speed of image acquisition is proportional to the number of SLM masks, and thus a better compressive sensing technique would decrease the image reconstruction time. On the other hand, a larger information capacity in each SLM mask also reduces the time and thereby improves the frame rate. Therefore, the proposed SLM with FMIM technique is very promising as a core component in the single pixel imaging module with a faster frame rate that could reach KHz frequency [40].

## 3. Discussion

The proposed chiral scheme enabled by bimorph cantilever offers an extremely simple artificial platform, and it is the simplified microhelix configuration that makes it possible to realize structural reconfiguration with a remarkably weak actuation voltage. The unique diatomic metamolecule that comprises two identical meta-atoms with opposite handedness enables a complete set of chiral conformations on a single platform. Such a reconfigurable metamolecule scheme avoids spatial upward and downward structural reversal that was commonly adopted to access a perfect chirality reversal between* D*- and* L*-conformations. Coexistence of 3D and 2D meta-atoms in a metamolecule provides a synchronized platform of dual-band intrinsic and extrinsic chirality, which provides profound insights into chiro-optical phenomenon. The four chiral conformations are reconfigurable in a programmable manner via dibit electrical encoding at a sub-MHz frequency, and the modulation performance could be further improved by adopting silicon based torsional actuators [42].

Although a dual-channel encoding logic of each pixel is applied in the proposed SLM scheme, it is in fact not necessary. Since only* D*- and* L*-conformations are valid for intensity modulation with [10] and [01] sequences, single-channel encoding logic is enough to reconfigure the diatomic metamolecule, which would largely decrease the complexity of electrical circuit design in field programmable gate array (FPGA), and thereby increase the pixel densities for a higher imaging resolution. The intensity extinction ratio of SLM depends on the polarization contrast between the two conformations, and the ideal scheme would be orthogonal output states (±45° rotation of polarization plane at* D*- and* L*-conformations). This would be realized by optimizing the geometrical parameters of microhelices such as the metal thickness.

In summary, we report an experimental study of dual-band chirality with intrinsic and extrinsic chiral responses by exploiting a simple cantilever based* diatomic* metamolecule. The paradigm enables abundant chiral conformations that are electrically programmable via dibit encoding with 10 V actuation voltage. The proposed chiral scheme provides huge potential for applications in efficient programmable polarization-based photonic devices with significant polarization contrast and rapid encoding speed. A transmission-type SLM is designed utilizing the programmable optical activity of the chiral scheme which is envisioned to be a promising component for terahertz single pixel imaging with a faster frame rate. This synthetic active chiral platform reveals exceptional flexibility in tailoring the ondemand properties. Electrically programmable polarization switch would immensely benefit terahertz communication for rapid polarization-division multiplexing and imaging. The low bias electrically reconfigurable chiral photonic scheme heralds a new paradigm towards construction of reconfigurable 3D constituents for stereoselective optical and terahertz technologies.

## 4. Materials and Methods

### 4.1. Sample Fabrication

A CMOS compatible process was adopted for the fabrication of the chiral devices. An 8^″^ silicon (Si) wafer was used as the substrate and 100 nm thick silicon oxide (SiO_2_) was deposited using low pressure chemical vapor deposition (LPCVD) process. This SiO_2_ layer acts as the sacrificial layer. After the sacrificial layer deposition, the first photolithography step was executed, and subsequently the SiO_2_ layer at the anchor region was dry-etched using reactive ion etching (RIE). Following this, we deposited a 50 nm aluminum oxide (Al_2_O_3_) layer using atomic layer deposition (ALD) process, followed by the sputter deposition of 400 (200, 600, 800, and 1000) nm thick aluminum (Al). Note that, at this stage, the bimorph layers (Al/Al_2_O_3_) at the anchor part were in physical contact with Si substrate and the remaining part was on top of SiO_2_ layer. Then the next photolithography step was carried out to define the resonator pattern along with the metallic interconnects and bondpads. Subsequently, Al and Al_2_O_3_ layers were both dry-etched, leaving the designed L-shaped bimorph pattern. Finally, vapor hydrofluoric acid (VHF) was used to isotropically etch the SiO_2_ sacrificial layer away, thereby suspending the cantilevers. The initial state of the released cantilevers was bent up due to the residual stress in the bimorph cantilevers, which is defined as “OFF” state. We note that the final VHF release process is not time-controlled which ensures high yield of devices. The mature fabrication procedure produces high quality and large-area uniform devices (see Figure 1 and [Supplementary-material supplementary-material-1]).

### 4.2. Measurements

A fiber laser based terahertz time-domain spectroscopy system was used to measure the transmission spectral response of devices and reference in dry nitrogen atmosphere at normal and tilted incidence (see [Supplementary-material supplementary-material-1]). Fourier transform was carried out to obtain the frequency domain spectra with both amplitude and phase information. The transmission amplitude spectra |*T*(*ω*)| and phase spectra *φ*(*ω*) were obtained by *T*(*ω*) = *T*^*Sam*^(*ω*)/*T*^*Ref*^(*ω*), where *T*^*Sam*^(*ω*) is the complex transmission signal of sample and *T*^*Ref*^(*ω*) is the complex transmission signal of reference, respectively.

### 4.3. Simulations

Numerical simulations (surface current distributions) were carried out using commercially available software (CST Microwave Studio) by a finite-element frequency domain solver with unit cell boundary conditions. Floquet ports with 18 modes were defined at the incident and receiving ports. Material parameters were obtained from the software library. Cantilevers were modelled as straight lines with a constant suspending angle which is variable depending on the aluminum thickness.

### 4.4. Calculations

The transmission coefficients (*T*_*ti*_) under the circular polarization base (*T*_*RR*_, *T*_*LR*_, *T*_*RL*_, and *T*_*LL*_) were retrieved from linearly polarized coefficients (*T*_*xx*_,* T*_*yx*_,* T*_*xy*_, and* T*_*yy*_, see [Supplementary-material supplementary-material-1] and [Supplementary-material supplementary-material-1]), where subscripts* i* and* t* denote the incident and transmitted light in a certain base. The* T* matrix of a new base is calculated by $\overset{-}{t}={\hat{\Lambda }}^{-1}\hat{T}\hat{\Lambda }\overset{-}{i}=\left(\begin{array}{cc} T_{11} & T_{12}\\ T_{21} & T_{22} \end{array}\right)\left(\begin{array}{c} {\overset{-}{i}}_{1}\\ {\overset{-}{i}}_{2} \end{array}\right)$ with $\hat{\Lambda }$ being the operator of the basis matrix and $\left(\begin{array}{cc} T_{11} & T_{12}\\ T_{21} & T_{22} \end{array}\right)$ the Jones matrix. Under the circular polarization base, the operator is $\hat{\Lambda }=\left(1/\sqrt{2}\right)\left(\begin{array}{cc} 1 & 1\\ i & -i \end{array}\right)$ and therefore transmission coefficients are described using the linear polarization base as follows:

(2) T^fcirc=TRRTRLTLRTLL=12·Txx+Tyy+iTxy−TyxTxx−Tyy−iTxy+TyxTxx−Tyy+iTxy+TyxTxx+Tyy−iTxy−Tyx

where* R* and* L* represent RCP and LCP, respectively.

Stokes parameters were introduced to numerically describe the output polarization states with* y*-polarized incidence as follows: *S*_0_ = |*T*_*xy*_|^2^ + |*T*_*yy*_|^2^, *S*_1_ = |*T*_*xy*_|^2^ − |*T*_*yy*_|^2^, *S*_2_ = 2|*T*_*xy*_||*T*_*yy*_|cos⁡*δ*, and *S*_3_ = 2|*T*_*xy*_||*T*_*yy*_|sin⁡*δ*, where *δ* is the phase difference (*δ* = *φ*_*yy*_ − *φ*_*xy*_). The polarization ellipse is calculated by tan⁡2*ψ* = *S*_2_/*S*_1_ and sin⁡2*χ* = *S*_3_/*S*_0_, where* ψ *and* χ* represent angle of polarization ellipse (AOP) and ellipticity, respectively. The ellipticity* χ* = 45° indicates a perfect RCP light, and* χ *= −45° indicates a perfect LCP light, and −45° <* χ* < 45° represents elliptically (*χ* = 0°, linearly) polarized light.

## Data Availability

All data is available in the main text or the Supplementary Materials.

## Supplementary Materials

Supplementary Materials Note S1. Mechanism of self-assembled MEMS cantilevers. Note S2. Evidence of intrinsic chirality at mode II. Note S3. Experimental setup and parameter retrieval. Note S4. Performance of the fabricated MEMS metadevice. Figure S1. Experimental evidence of intrinsic chirality. Figure S2. Experimental setup and strategy for measurements. Figure S3. Measured transmission spectra and surface current distributions. Figure S4. Polarization ellipse based on the measured ellipticity and AOP. Figure S5. Deformation profiles of cantilevers. Figure S6. Modulating output polarization state by adjusting the metal thickness of cantilevers. Movie S1. Characterization of the pull-in voltage of the metadevice. Movie S2. Microscopic image showing the uniform actuation of all the resonators in the large-area metadevice.
