# Supplementary material for: Electrically Programmable Terahertz Diatomic Metamolecules for Chiral Optical Control
Source: Research (Wash D C). 2019 Feb 27;2019:7084251. doi: 10.34133/2019/7084251 (PMC6750089; doi:10.34133/2019/7084251)
Supplement: Supplementary Materials — Note S1. Mechanism of self-assembled MEMS cantilevers. Note S2. Evidence of intrinsic chirality at mode II. Note S3. Experimental setup and parameter retrieval. Note S4. Performance of the fabricated MEMS metadevice. Figure S1. Experimental evidence of intrinsic chirality. Figure S2. Experimental setup and strategy for measurements. Figure S3. Measured transmission spectra and surface current distributions. Figure S4. Polarization ellipse based on the measured ellipticity and AOP. Figure S5. Deformation profiles of cantilevers. Figure S6. Modulating output polarization state by adjusting the metal thickness of cantilevers. Movie S1. Characterization of the pull-in voltage of the metadevice. Movie S2. Microscopic image showing the uniform actuation of all the resonators in the large-area metadevice. [file 7084251.f1.zip › 7084251.f1/Supplementary Materials_Cong et al.docx]

Supplementary Materials for Electrically programmable terahertz diatomic metamolecules for chiral optical control

**Authors**

Longqing Cong,1,2 Prakash Pitchappa,1,2 Nan Wang,3 Ranjan Singh1,2*

**Affiliations**

1Division of Physics and Applied Physics, School of Physical and Mathematical Sciences, Nanyang Technological University, Singapore 637371, Singapore.

2Centre for Disruptive Photonic Technologies, The Photonics Institute, Nanyang Technological University, 50 Nanyang Avenue, Singapore 639798, Singapore.

3Institute of Microelectronics, 11 Science Park Road, 117685, Singapore

*Correspondence to: [ranjans@ntu.edu.sg](mailto:ranjans@ntu.edu.sg)

**This file includes:**

Supplementary Text

Figs. S1 to S6

Captions for Movies S1 to S2

**Other Supplementary Materials for this manuscript include the following:**

Movies S1 to S2

Supplementary Text

**Note S1: Mechanism of self-assembled MEMS cantilevers**

Micro-electro-mechanical system (MEMS) is modelled as a parallel plate capacitor with capacitance C, , where *A* is the area of the plate, *ε* is the dielectric constant of the medium between the plates, and *g* is the gap distance between them. For a variable capacitor, the capacitance is calculated by . The energy of the capacitor is given by with applied voltage V, and thus the electrostatic force between the plates is determined by . Assuming the movable plate is attached to a spring with constant *K*, the equilibrium point at the given voltage is described by  without considering the gravity force. According to this equation, there will be a voltage where there is no solution of *z*, and this voltage is the so-called pull-in voltage. When the drive voltage reaches the pull-in voltage, the displacement leaps to close the gap; and when the voltage is reduced, the plate remains in contact with the counter electrode as long as the electrostatic attractive force is greater than the mechanical restoring force. The pull-in phenomenon will occur when the derivative of voltage with respect to the position *z* is zero, that is , from which we get the pull-in distance and the corresponding pull-in voltage . Therefore, usually “ON” and “OFF” states by pull-in voltage are applied for the MEMS design sitting on the substrate where the substrate is connected to ground and the voltage is applied to the suspended cantilevers.

**Note S2: Evidence of intrinsic chirality at mode II**


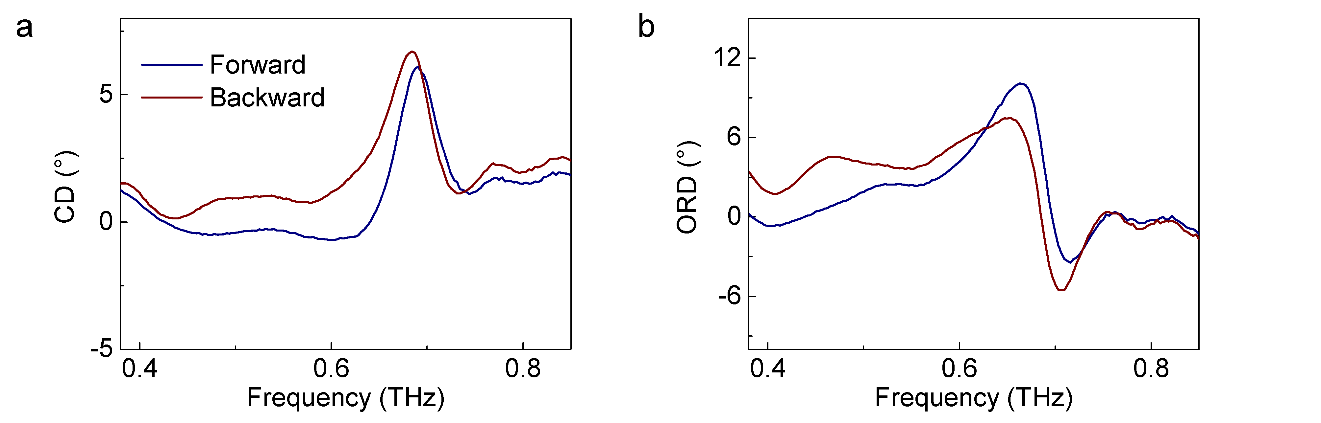


**Fig. S1. Experimental evidence of intrinsic chirality.** The polarity of CD and ORD will not be reversed for intrinsic chirality when sample is shined from front and back at normal direction.

**Note S3: Experimental setup and parameter retrieval**


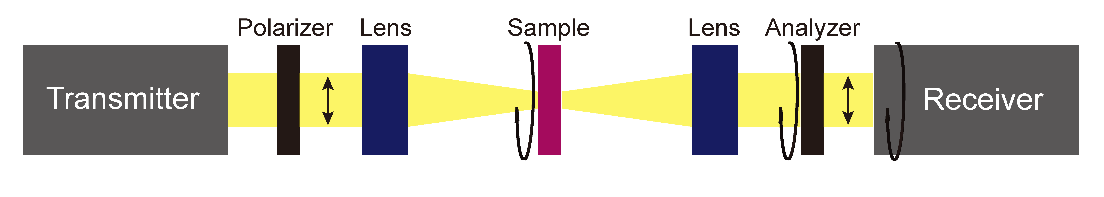


**Fig. S2. Experimental setup and strategy for measurements of four elements of Jones matrix.**

**Four Jones matrix measurements:** the fiber laser based terahertz time-domain spectroscopy system enables the experimental flexibility for polarization related measurements with the experimental setup as schematically illustrated in Fig. S2. The transmitter was fixed with a polarizer to ensure pure linear polarization state. The co- and cross-polarized transmission components after samples were collected via rotating analyzer and receiver synchronously by 90°. The transmission response of samples with orthogonal polarization incidence was obtained by rotating the sample by 90°. This approach reduced the complexity of measurements and thus minimized the possibility of introducing measurement errors. However, it is specially noted that *an extra relative 180°* *phase accumulation* was introduced between the two cross-polarized components (T*yx* and T*xy*) due to the Pancharatnam-Berry phase (or geometric phase due to the 90° rotation of samples). At the stage of data processing, this extra geometric phase had to be removed. The measured linear polarization transmission spectra are shown in Fig. S3a at four conformations for sample with metal thickness of 400 nm. Co-polarized transmission spectra (T*xx* and T*yy*) reveal different features due to the presence of periodic anchor wires that act as a grating at all four conformations. Zero cross-polarized components (T*yx* and T*xy*) were measured at *a*- and *r*-conformations, indicating no polarization rotation and modulation. However, large intensity of cross-polarized components emerges with *D*- and *L*-conformations, which gives rise to the modulation of output polarization states.


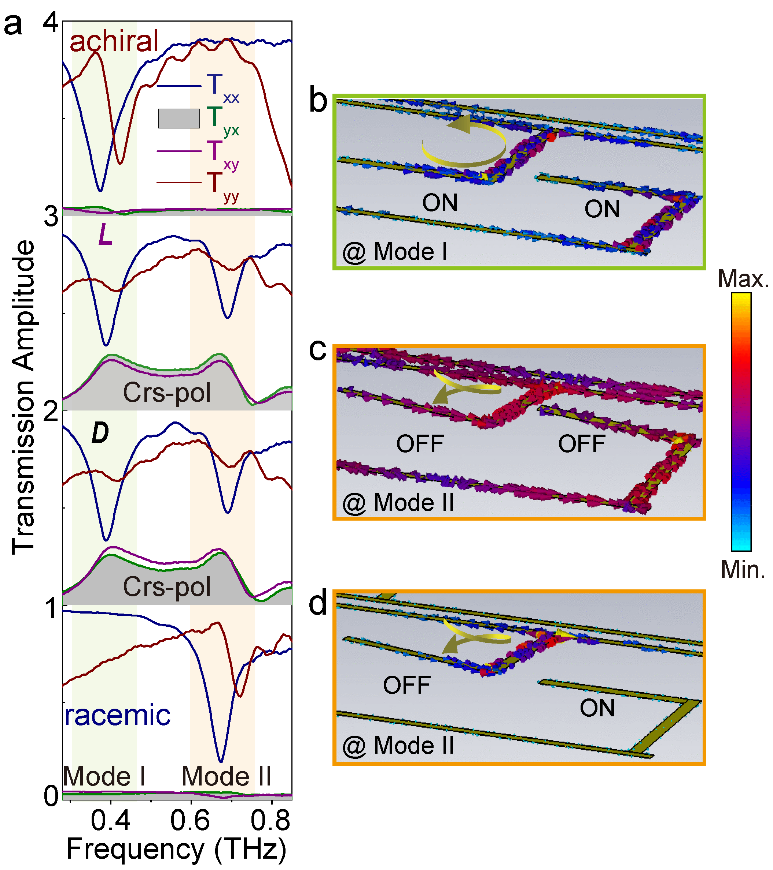


**Fig. S3. Measured transmission spectra and surface current distributions. (A)** Measured transmission spectra of four elements in Jones matrix with linearly polarized incidence for four chiral conformations. **(B)** Surface current distribution of achiral conformation at Mode I. **(C, D)** Surface current distributions of racemic and *L*-conformation at Mode II.

**Simulated surface current distributions:** At *a*-conformation (Fig. S3b), each L-shape cantilever together with anchor form a 2D split ring resonator, and surface current oscillating on the 2D resonator at Mode I induces a magnetic dipole that is orthogonal to net in-plane electric dipole. As for *r*-conformation in Fig. S3c, similar current loop is observed on an individual microhelix at Mode II, but are guided to flow along the 3D conformation. Such a 3D current flow would give rise to electric and magnetic dipole interaction, and thus induce intrinsic chirality. The intrinsic chirality would manifest as nontrivial CD and ORD in the far field in the scenario of *L*- (Fig. S3d) or *D*-conformation. However, the intrinsic chirality is cancelled with each other due to the opposite polarity of neighboring metaatoms with symmetric geometric parameters in a metamolecule, and thus nontrivial CD and ORD are captured in the far field.

**Output polarization states measurements at polar coordinate:** output polarization states were measured by rotating the analyzer by 360° with a step of 10° while keeping sample and all other components fixed. After Fourier transform from time-domain signals, angle-resolved frequency domain amplitude spectra (*Tangle*) were obtained after normalization with spectra of substrate in dry nitrogen atmosphere. Angle-resolved intensity spectra were calculated by , and is the relative polarization angle between analyzer and receiver where indicates the parallel scenario. We note that two points at were ignored due to the limitation of this experimental stratagy, which do not affect the results as shown in Fig. 5E. We plotted the angle-dependent intensity of output light at the specified frequencies in a polar coordinate to visualize the polarization states.


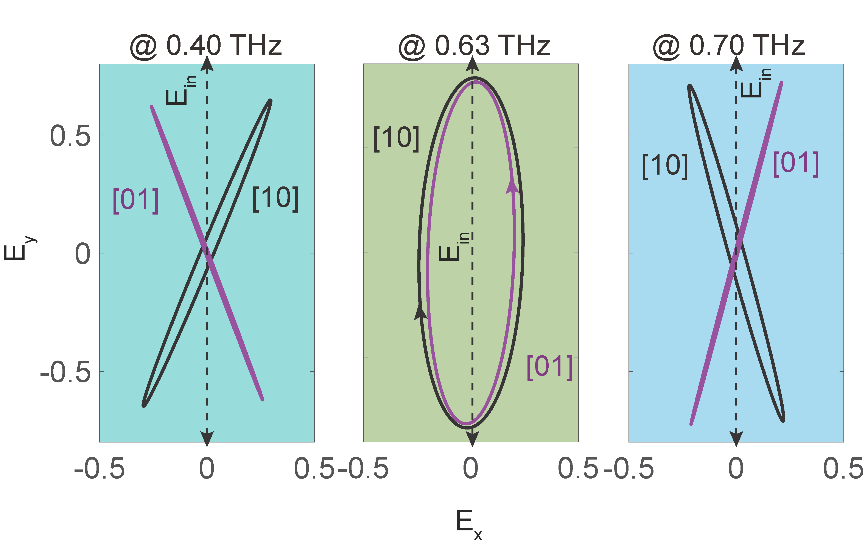


**Fig. S4 Polarization ellipse based on the measured ellipticity and AOP.** At 0.40 and 0.70 THz, large rotation of polarization plane is induced without strong modulation of incident polarization states (linear polarization). The polarization rotation angles are opposite with [10] and [01] inputs. At 0.63 THz, large modulation of polarization state is induced without large rotation of orientation of polarization plane. For [10] and [01] inputs, handedness of output polarization ellipse is opposite.

**Note S4: Performance of the fabricated MEMS metadevice**

**Fig. S5 Deformation profiles of cantilevers.** Simulated deformation profiles of the (A) continuous wire resonator (CWR) and (B) split ring resonator (SRR) cantilevers at the fundamental resonance frequency.

The profile of suspended micro cantilevers for continuous wire resonator (CWR) and split ring resonator (SRR) were measured using Lyncee Tec. reflection digital holographic microscope (R-DHM). The released device chips were wire bonded to a PCB with one voltage power supply for actuating the CWR cantilevers and the other for SRR cantilevers, respectively. Silicon substrate was kept at ground potential and the cantilevers were positively biased. The cantilevers do not snap down completely onto the Si substrate until the applied voltage exceeds a specific voltage (pull-in voltage, 10 V). The dielectric Al2O3 layer beneath the Al layer ensures no current flowing from Al layer to Si substrate at the pull-in state. This is crucial since current flow through the cantilevers would raise up the temperature locally, and thereby cause permanent damage to the device by thermally melting the Al tips. At different stages of applied voltages, the cantilever profile can be measured directly from the R-DHM unit and the electrical isolation between the CWR and SRR allows for independent reconfigurations of CWR and SRR cantilevers.

The switching speed is limited by the resonance frequency of the respective cantilevers which were simulated using Finite Element Modelling. The simulated fundamental mechanical resonance frequency of continuous wire resonator (CWR) cantilevers was estimated to be ~164 kHz and that of a half square ring resonator (SRR) cantilevers was estimated to be ~486 kHz. The displacement profiles of the CWR and SRR micro cantilevers as shown in Fig. S5 confirm the first order resonance frequency for the respective cantilevers. However, there is large contact area between the cantilevers and substrate when the cantilevers are switched to the “ON” state, which causes strong adhesion force and hence adversely affect the switching speed of the device. The adhesion force is an interface phenomenon that largely depends on the interface quality, surface roughness, and contact area. An alternative method is being explored to improve the switching speed, uniformity and reliability of the MEMS based metadevice employing torsional actuators.


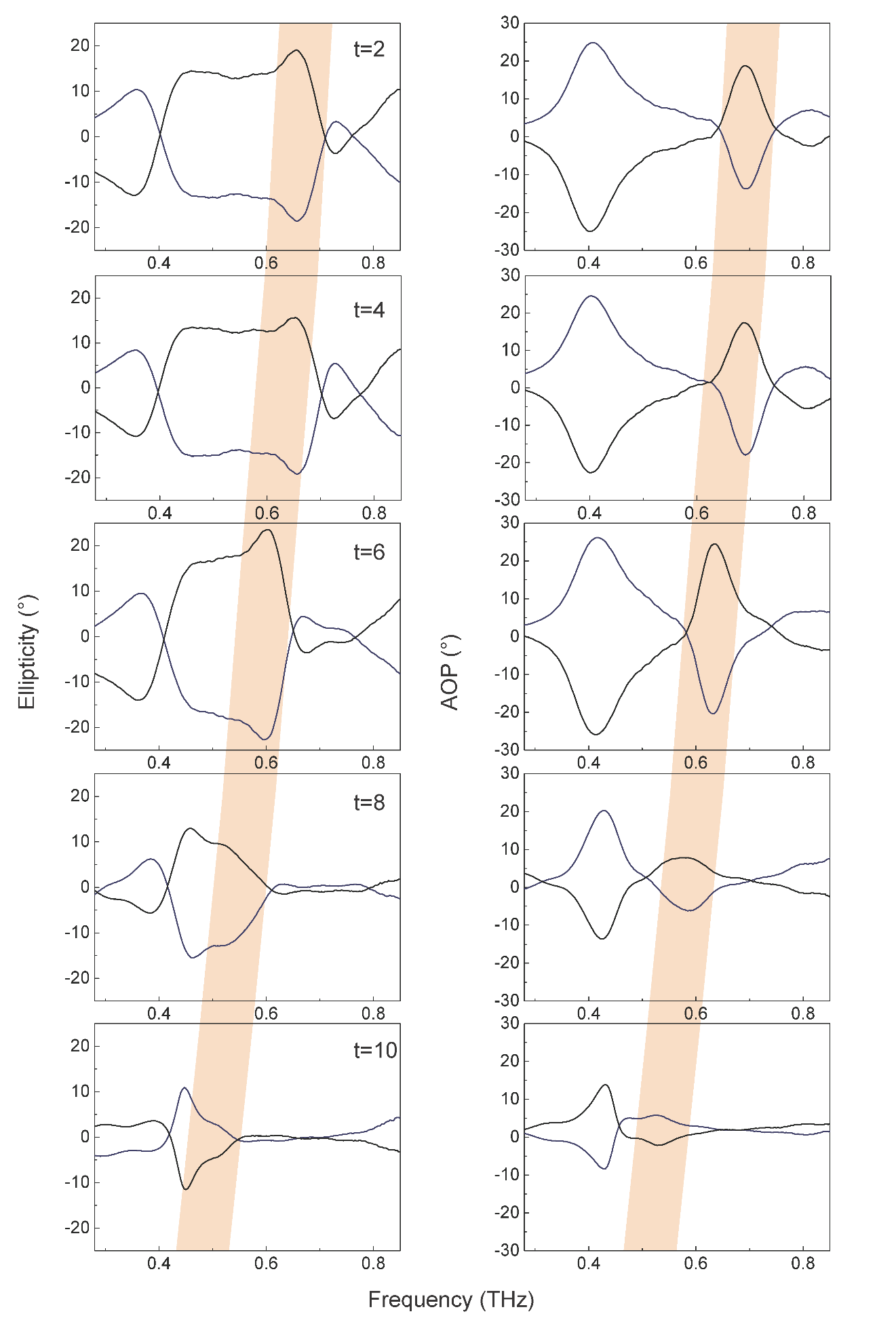


**Fig. S6.** Modulating output polarization state by adjusting the metal thickness of cantilevers. Output polarization ellipticity, rotation angle, and operating frequencies are readily modulated for on-demand application requirements.

**Movie S1.** Characterize the pull-in voltage of the metadevice by increasing actuation voltage from 0 to 10 V. We can observe that most of the unit cells are pull-in at actuation voltage of 9 V, and all the unit cells are fully pulled back on the substrate with 10 V actuation voltage. The metadevice was encoded with [10] sequence in this test.

**Movie S2.** Microscopic image showing the uniform actuation of all the resonators in the large-area (10×10 mm2) metadevice array at dextrorotatory conformation.
